# Children and Adolescents Treated for Valvular Aortic Stenosis Have Different Physical Activity Patterns Compared to Healthy Controls: A Methodological Study in a National Cohort

**DOI:** 10.1007/s00246-021-02540-1

**Published:** 2021-02-01

**Authors:** Pia Skovdahl, Cecilia Kjellberg Olofsson, Jan Sunnegårdh, Jonatan Fridolfsson, Mats Börjesson, Sandra Buratti, Daniel Arvidsson

**Affiliations:** 1 Center for Health and Performance, Department of Food and Nutrition, and Sport Science, Faculty of Education, University of Gothenburg, Gothenburg, Sweden; 2 Department of Pediatrics, Institute of Clinical Sciences, Sahlgrenska Academy, University of Gothenburg, Gothenburg, Sweden; 3 Department of Pediatrics, Sundsvall Hospital, Sundsvall, Sweden; 4 Department of Cardiology, The Queen Silvia Children’s Hospital, Sahlgrenska University Hospital, Gothenburg, Sweden; 5 Center for Health and Performance, Department of Molecular and Clinical Medicine, Sahlgrenska Academy, University of Gothenburg, Gothenburg, Sweden; 6 Sahlgrenska University Hospital/Östra, Gothenburg, Sweden; 7 Department of Psychology, University of Gothenburg, Gothenburg, Sweden

**Keywords:** Accelerometer, Adolescents, Children, Congenital heart defect, Physical activity pattern

## Abstract

Previous research in children and adolescents with congenital heart defects presents contradictory findings concerning their physical activity (PA) level, due to methodological limitations in the PA assessment. The aim of the present cross-sectional study was to compare PA in children and adolescents treated for valvular aortic stenosis with healthy controls using an improved accelerometer method. Seven-day accelerometer data were collected from the hip in a national Swedish sample of 46 patients 6–18 years old treated for valvular aortic stenosis and 44 healthy controls matched for age, gender, geography, and measurement period. Sports participation was self-reported. Accelerometer data were processed with the new improved Frequency Extended Method and with the traditional ActiGraph method for comparison. A high-resolution PA intensity spectrum was investigated as well as traditional crude PA intensity categories. Children treated for aortic stenosis had a pattern of less PA in the highest intensity spectra and had more sedentary time, while the adolescent patients tended to be less physically active in higher intensities overall and with less sedentary time, compared to the controls. These patterns were evident using the Frequency Extended Method with the detailed PA intensity spectrum, but not to the same degree using the ActiGraph method and traditional crude PA intensity categories. Patients reported less sports participation than their controls in both age-groups. Specific differences in PA patterns were revealed using the Frequency Extended Method with the high-resolution PA intensity spectrum in Swedish children and adolescents treated for valvular aortic stenosis.

## Introduction

The life expectancy for children and adolescents born with a congenital heart defect (CHD) has radically improved the last decades [1, 2]. This development has formed a relatively new patient group. Prior research in the general population has established the positive effects of physical activity (PA) on cardiovascular health, psychosocial health, self-esteem, learning, motor-skill development, and quality of life [3], with high-intensity PA appearing as particularly beneficial to health [4]. Physical limitations of the cardiovascular system [5, 6], restrictions from parents and caregivers [7, 8], and low self-efficacy [7] have been emphasized by prior research as factors that prevent PA participation in children and adolescents with CHD. Consequently, it may be assumed that they are less physically active than their healthy peers.

Congenital valvular aortic stenosis (VAS) occurs in 3–5% of children with CHD. The severity of the defect varies from life-threatening condition at birth (critical aortic stenosis in the neonate) to a condition with need for intervention later in life. Treatment during childhood aims to preserve the native aortic valve with either surgical valvotomy or balloon valvotomy, but often with a need of aortic valve replacement at a later stage. Apart from the life-threatening situation in the neonate with critical valvular aortic stenosis, a mean Doppler gradient of 50 mmHg or more is the main indication for treatment. In Sweden, the primary treatment is surgical valvotomy. As VAS is a complex and lifelong disease where re-interventions and further surgery is common [9], patients often show residual or acquired abnormalities of the left heart structure which might affect cardiovascular capacity [10]. Even if exercise function is preserved in most patients treated for VAS in infancy, there may still be reduced peak VO_2_ in some individuals, probably reflecting inability to increase stroke volume [6].

As the positive effects of PA is well acknowledged, it is important to establish the habitual PA levels in children and adolescents with VAS and other CHDs as part of the clinical assessment and to provide individually adapted prescriptions [11–13]. Exercise restrictions have traditionally been recommended for some patients with VAS because of a perceived increased risk for sudden death. In the current era, sudden unexpected death during exercise in this patient group is uncommon [14, 15], probably due to a more active approach to treatment than in the earlier era of pediatric cardiology. Still, many patients with VAS might be advised to avoid strenuous PA [11–13].

Uncertainties exist regarding the PA levels of children and adolescents with CHD, as prior studies show contradicting results and in general have pronounced methodological limitations and diversities [16]. The quantification of PA relies primarily on objective methods, with the accelerometer being the most frequently used one [17], as subjective methods typically possess poor validity and reliability in children. However, great irregularities have been observed in data collection, value calibration, and data processing in accelerometer-based studies, making comparisons of results difficult [17]. Moreover, the accelerometer method commonly used in previous research in CHD, the ActiGraph counts (AG), shows difficulties in capturing intermittent and high-intensity PA [4, 18, 19], which have restricted correct classification of PA [20]. These measurement errors are more prominent in children due to their movement patterns compared to in older individuals. Recent methodological developments have improved assessment of PA [4, 18–21] and the association with cardiometabolic health [22]. These improvements may help to clarify the uncertainties regarding the PA level of children and adolescents with CHD.

The aim of the present study was to compare PA patterns between children and adolescents treated for VAS and healthy controls using new improved accelerometer assessment with the Frequency Extended Method (FEM) [18–21] and a detailed spectrum of PA intensities [22].

## Methods

### Design

The present cross-sectional study consists of two parts. The main study collected free-living accelerometer data in children (6–12 years) and adolescents (13–18 years) with VAS and in healthy controls matched for age, sex, and geography. Data collection was performed during the same week in patient and control in order to control for seasonal variation in PA. To be able to evaluate the contribution of the new FEM, we also performed the analyses using the traditional AG method for comparison. We primarily analyzed the processed accelerometer data in detailed spectrum of PA intensities as recently being recommended [4]. In order to facilitate interpretation of the PA data in accordance to previous research, a calibration study was included to provide traditional crude PA intensity categories applied on the free-living data. Ethical approvals have been achieved by the Regional Ethics Committee in Gothenburg, No. 582-18 (main study) and No. 1026-17 (calibration study). All participants and/or parents provided consent to participate.

### Main Study

#### Sample

All patients treated for isolated VAS in Sweden aged 6–18 years old were identified in surgical registers and the Swedish Registry of Congenital heart Disease (SWEDCON). 121 patients (female 21.5%) with treated VAS, born between 2001 and 2013, were identified. Age at first treatment was < 30 days in 40%, 1 month to 1 year in 30% and older than 1 year in 30%. Healthy controls matched for age, sex, and geography were generated from Statistics Sweden (www.scb.se), five controls per patient considering the risk for low participation rate. All participants were asked if any severe health issues were present that restricted the possibility of being physically active. No participant was excluded due to this reason. One patient willing to participate was excluded due to recent surgical intervention. Fifty-two patients (female 21.2%) and 50 controls (female 20.0%) answered and agreed to participate.

#### Protocol

All data were collected September 2019 to March 2020. The triaxial accelerometer Axivity AX3 (Axivity Ltd, UK) was delivered by mail with age-customized instructions. Participants were instructed to wear the sensor continuously for seven consecutive days over the right hip in an elastic belt around the waist. A diary was provided to capture sleep-time, sports/activity type, and information of normal day. The OmGUI software (Axivity Ltd., Newcastle upon Tyne, UK) was used for accelerometer initialization and data extraction, with a sampling frequency of 50 Hz and 8 g sensitivity. Output from the three axes were combined to a vector magnitude and resampled to 30 Hz. Data were processed to the output mean mg of 3 s epochs. This epoch-length captures the intermittent and sporadic movement pattern in children [4].

The original AG method uses a narrow frequency filter (range 0.29–1.63 Hz) excluding acceleration data from higher movement frequencies and PA intensities [18]. As shorter individuals move with higher frequency than taller, the AG filter excludes more of the acceleration data in children compared to older individuals [18–20]. The new FEM filter includes a wider frequency range (0.29–10.0 Hz), which captures all relevant accelerations generated at the hip. Both methods were applied to the accelerometer data.

Participants that provided data for ≥ 4 days of measurement (≥ 3 weekdays and ≥ 1 weekend day) with a valid day criterion set to ≥ 10 h-a-day were included in the analyses. Night-time was removed in correspondence with diary annotations. Non-wear time was defined as 60 min of uninterrupted zeros and allowance for < 2 min of exception. The final sample consisted of 46 patients (female 19.6%) and 44 controls (female 20.4%).

#### Output Variables and Statistical Calculations

Two different PA outputs are presented: (1) time spent across a high-resolution PA intensity spectrum, dividing the PA intensity range into 22 intervals (bins); and (2) time spent in traditional PA intensity categories defined from the calibration study: sleep (reported in diary), sedentary time (SED), light PA (LPA), moderate PA (MPA), vigorous PA (VPA), and very vigorous PA (VVPA). In addition, the proportion reaching WHO recommendation of ≥ 60 min per day of MVPA was determined [23], applying both a less strict criterium of an average of ≥ 60 min per day, and a stricter criterium of ≥ 60 min 6 out of 7 days (i.e., most days) [24]. Mean group difference and bootstrapped 95% confidence interval were determined across the PA intensity spectrum. Two-tailed *t* tests for independent groups were performed for each traditional PA intensity category, as well as for group characteristics and reported sports participation. Chi-square was used to test differences in frequency distributions. Analyses were performed in Matlab 2020a (MathWorks, Natick, MA, USA).

### Calibration Study

#### Sample

Calibration data were previously collected in 2018 in our research lab from 10 children 9–11 (3 females, 7 males) and 10 adolescents 14–16 (5 females, 5 males) years old [19]. They were recruited through local sports clubs, institutional staff, and personal contacts by oral information and by written announcements through email.

#### Protocol

Oxygen uptake was collected with Oxycon Pro (Jaeger, BD Corporation, Franklin Lakes, NJ, USA) and accelerometer data with Axivity AX3 (Axivity Ltd, UK) during sitting at rest, standing, and walking (3, 4, 5, 6 km h^−1^) and running (8, 10, 12 km h^−1^) on a treadmill (26). To be able to compare PA intensity between age-groups, the VO_2net_ (VO_2total_ − VO_2stand_, ml kg^−1^ min^−1^) was used as criterion measure [21]. When two individuals from different age-groups achieve the same VO_2net_, they perform the PA with the same metabolic effort, but the younger and shorter individual children are moving at a slower speed and producing less acceleration than the older and taller one. The metabolic equivalent of task (MET; VO_2total_/VO_2rest_) has traditionally been used as criterion measure, but is not comparable between ages [21].

Accelerometer data were processed with the FEM and the AG method as in the main study. Smoothing splines were fitted to the accelerometer output and VO_2net_ to set accelerometer cut-points for LPA, MPA, VPA, and VVPA. To further facilitate interpretation, we also present PA intensity in relation to the MET applying 1.5, 3.0, 6.0, and 9.0 METs as cut-points for LPA, MPA, VPA, and VVPA, as well as to movement speed (km h^−1^). The MPA cut-point from VO_2_net was used to determine compliance to the WHO PA recommendation.

## Results

### Main Study

Group characteristics are presented in Table 1. Patients reported statistically significant less sports participation than the controls in both age-groups.

**Table 1:** Group characteristics

Variable	Children 6–12 years			Adolescents 13–18 years		
	VAS (*n* = 27)	Controls (*n* = 29)	*p*-value	VAS (*n* = 19)	Controls (*n* = 15)	*p*-value
Age (years), mean (SD)	9.8 (2.6)	9.7 (2.0)	0.86	15.9 (2.1)	15.1 (2.0)	0.86
Gender, female, n (%)	2 (7.4)	5 (18.5)	0.27	7 (36.8)	4 (21.0)	0.53
Sports participation (times/week), mean (SD)	2.1 (1.4)	3.2 (1.6)	0.009	2.8 (2.2)	4.2 (2.4)	0.009
Valid days, mean (SD)	6.9 (0.3)	6.9 (0.3)	0.94	6.8 (0.4)	6.7 (0.5)	0.94
Wear time (%), mean (SD)	98.7 (2.1)	97.6 (3.3)	0.15	97.5 (4.5)	97.7 (5.7)	0.15

**Fig. 1:**
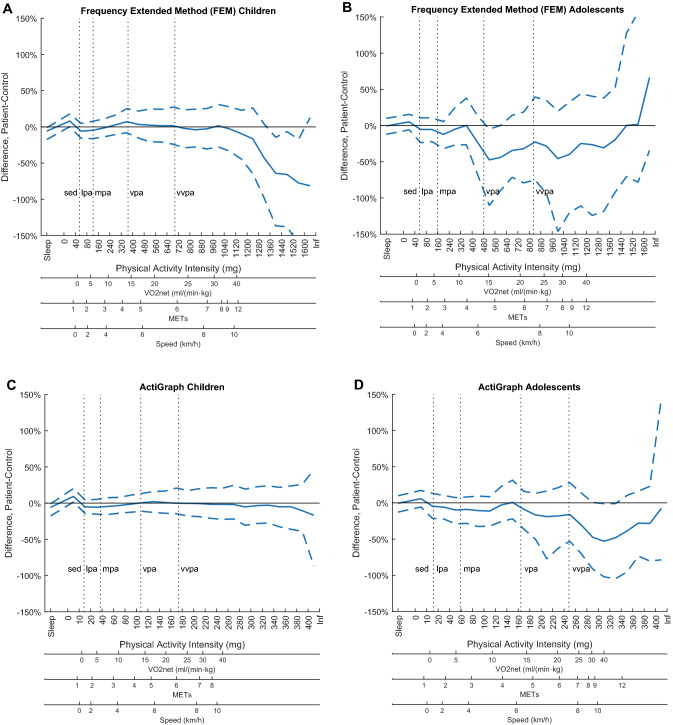
**a**–**d** Mean (bootstrapped 95% CI) difference (%) between patients and controls in time spent in each PA intensity interval (bin) across the intensity spectrum (acceleration, mg); **a** Frequency Extended Method in children, **b** Frequency Extended Method in adolescents, **c** ActiGraph method in children, **d** ActiGraph method in adolescents. VO_2_net, METs and movement speed from the calibration study are provided to support interpretation of the PA intensity performed. Data from the main study

**Fig. 2:**
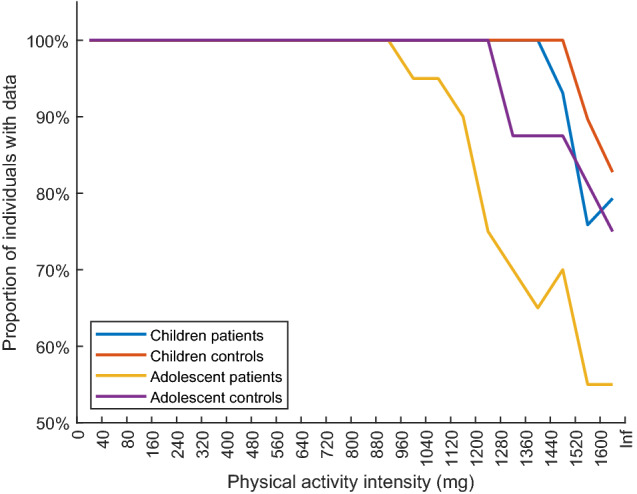
Proportion of individuals in each group having data across the physical activity intensity spectrum. Data from the main study

The FEM with the detailed PA intensity spectrum analysis revealed that the children treated for VAS had a pattern of less PA at the very highest intensity spectra compared to their controls, with intensities where the 95% confidence interval did not overlap zero difference (Fig. 2a). With the cruder PA intensity categories, this pattern was less evident with no statistically significant differences apparent (Table 2). Further, the PA intensity spectrum indicated a pattern of more SED in the patients than in the controls (Fig. 1a), reaching an average difference of 44 min day^−1^ between the groups in this category (Table 2), but with no statistically significant differences identified. With the less strict criterium, all of the patients and the controls reached the WHO PA recommendation. With the stricter criterium, 93% of the patients and 93% of the controls reached the PA recommendation.

**Table 2:** Mean time spent in physical activity intensity categories by age-group and accelerometer method

Time, min day^−1^	Children 6–12 years								Adolescents 13–18 years							
	FEM				AG method				FEM				AG method			
Category	VAS (*n* = 29)	Controls (*n* = 30)	Diff	*p*-value	VAS (*n* = 29)	Controls (*n* = 30)	Diff	*p*-value	VAS (*n* = 23)	Controls (*n* = 16)	Diff	*p*-value	VAS (*n* = 23)	Controls (*n* = 16)	Diff	*p*-value
Reported sleep	588.7	624.0	− 35.3	0.11	588.7	624.0	− 35.3	0.11	535.7	549.9	− 14.2	0.61	535.7	549.9	− 14.2	0.61
SED	518.6	474.6	44.0	0.06	501.2	455.8	45.4	0.054	640.6	606.7	33.9	0.34	634.6	598.9	35.7	0.32
LPA	149.2	157.9	− 8.7	0.29	100.2	105.5	− 5.3	0.30	149.8	155.3	− 5.5	0.68	117.7	123.7	− 6.0	0.57
MPA	144.2	144.2	0.0	1.00	139.1	143.3	− 4.2	0.60	101.6	110.4	− 8.8	0.46	118.1	126.6	− 8.5	0.50
VPA	29.0	28.0	1.0	0.74	67.7	67.1	0.6	0.90	7.5	11.6	− 4.1	0.049	24.2	27.8	− 3.7	0.39
VVPA	10.3	11.3	− 1.0	0.53	43.2	44.3	− 1.1	0.81	4.7	6.1	− 1.4	0.44	9.7	13.0	− 3.3	0.23
MVPA	183.5	183.5	0.0	1.00	249.9	254.7	− 4.7	0.76	113.8	128.1	− 14.2	0.32	152.0	167.4	− 15.4	0.36
VPA + VVPA	39.3	39.3	0.0	1.00	110.9	111.4	− 0.5	0.96	12.2	17.6	− 5.4	0.13	33.9	40.9	− 7.0	0.29

Physical activity intensity categories defined by VO_2_net cut-points

SED, sedentary physical activity; LPA, light physical activity; MPA, moderate physical activity; VPA, vigorous physical activity; VVPA, very vigorous physical activity; MVPA, moderate-to-vigorous physical activity; VPA + VVPA, Vigorous-to-very-vigorous physical activity

In the adolescents with the FEM, the detailed PA intensity spectrum indicated a pattern of less PA across the higher PA intensity spectra in the patients than in the controls, although there was a switch at the very highest intensity level (Fig. 1b). None of these differences were statistically significant. The cruder PA intensity classification confirmed this tendency, with statistically significant less time in VPA in the patients (Table 2). 95% of the patients and 94% of the controls reached the recommended amount of PA with the less strict criterium, and 54% and 56%, respectively, with the stricter criterium.

Figure 2 complements previous results and presents the proportion of individuals having accelerometer data across the PA intensity spectrum in each of the four groups using the FEM. It visualizes two characteristics of the data collected: first, the lower amount of time spent in the higher intensity spectra by the patients compared to the controls, especially among the adolescents; second, the decreasing proportion of individuals having data at the higher intensity spectra, affecting the possibility of detecting statistically significant group differences.

With the AG method in the children, the detailed PA intensity spectrum did not demonstrate the pattern of less PA at the highest PA intensity spectra in the patients compared to the controls (Fig. 1c). The patients spent 45 min day^−1^ more time in SED than the controls, but this difference was not statistically significant (Table 2).

A similar pattern of difference in PA between adolescent patients and controls was seen with the AG method as with the FEM, but the largest relative difference was found at even higher PA intensity spectra with the AG method where the 95% confidence interval did not overlap zero difference (Fig. 1d), which was confirmed with the cruder PA classification (Table 2). No statistically significant differences between adolescent patients and controls were identified for the cruder intensity classification with the AG method.

The larger 95% confidence interval demonstrated with the FEM compared to the AG method for both age-groups (Fig. 1a and c versus b and d) indicates that the FEM captured more of the actual movements performed and consequently larger inter-individual variation in PA.

### Calibration study

**Fig. 3:**
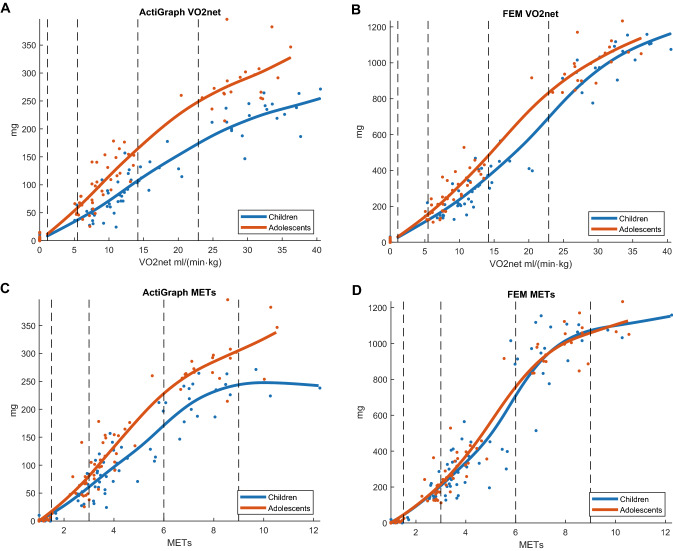
**a**–**d** VO_2_net plotted against accelerometer absolute PA intensity (acceleration, mg) for **a** the ActiGraph method and **b** Frequency Extended Method (FEM); MET plotted against accelerometer absolute PA intensity (acceleration, mg) for **c** the ActiGraph method and **d** the Frequency Extended Method (FEM). The accelerometer cut-points for LPA, MPA, VPA, and VVPA (vertical dashed lines) are determined from smoothing splines modeling. Data from the calibration study

With the AG method, children and adolescents are separated with a distinct suppression of the accelerometer output at higher intensities (Fig. 3a). With the FEM, the separation of the children and adolescents is less distinct with no suppression of the accelerometer output (Fig. 3b). Modeling of accelerometer PA intensity versus MET is displayed for comparison (Fig. 3c, d). Table 3 provides the calibrated PA intensity cut-points for the FEM and the AG method. Children show lower cut-points for the respective PA intensity category than the adolescents. This is because they are moving at a slower speed and producing less acceleration, when the cut-points are set at the same VO_2net_ values representing similar metabolic effort.

**Table 3:** Calibrated cut-points (mg) for physical activity intensity categories by age-group and accelerometer method

Unit: mg	Children 6–12 years		Adolescents 13–18 years	
Category	FEM	AG method	FEM	AG method
SED	< 29	< 8	< 32	< 12
LPA	29	8	32	12
MPA	124	37	157	59
VPA	368	108	482	165
VVPA	695	174	830	249

## Discussion

The main result of the present study is that the application of the improved FEM with the detailed PA intensity spectrum revealed differences in PA patterns that were less evident using the traditional accelerometer method with its measurement errors. The children treated for VAS had a pattern of less PA at the highest PA intensity spectra and more SED than their controls, while the adolescents treated for VAS tended to have less PA of higher intensities overall and more SED.

The PA performed at the highest intensity spectra by children is considered to include intermittent bursts, typically sustained in the inborn movement pattern of children when doing different sports activities [4]. The pattern with lower PA in children treated for VAS compared to their healthy peers in the present study may indicate that this type of activity is restrained in this patient group. The less frequent sports participation in children with VAS compared to their controls further supports the findings from the accelerometer assessment using the FEM. A previous study of Swedish children with CHD also reports less sports participation than in their healthy peers [25]. The reason why this accelerometer PA pattern was not visible with the crude classification is because the total volume of time spent at the highest PA levels is relatively small, mainly containing seconds rather than minutes in children. One may speculate on how this reduced intermittent moment pattern affects further on participation in activities and sports. The tendency of less PA of higher intensity overall in the adolescents treated for VAS may indicate a major behavior change, in addition to what is observed in the general population [26]. A relevant question is whether the PA pattern in adolescents treated for VAS is a consequence of the behavioral pattern observed in the children treated for VAS. To confirm this transfer of behavior, a longitudinal study design is required.

Previous research using accelerometers for the assessment of PA in children and adolescents with CHD has shown contradicting results, possessing great variances in sample size and characteristics of the studied CHD, in data collection and processing settings, using the AG method [16]. Consequently, the varying results may be caused by numerous possible measurement errors, as the results presented are highly reliable on the methodology used even if displayed similarly [17]. As an illustration, epoch-lengths ranging from 3 to 60 s (or not-reported) have been observed in these studies. Different epoch-lengths lead to significant variations in time spent in the different PA intensities [4]. Shorter epochs have been recommended, especially when studying children whose movement pattern mainly consists of intermittent, sporadic bursts [4].

Further, misclassification of PA with the traditional AG method has been reported as being low at the lighter intensities (1–2%) and large at the higher intensities (> 90%) when compared to wider filters [20]. Therefore, there was little difference between the FEM and the AG method in the assessment of SED in the present study. However, with a classification agreement of only approximately < 10% at VPA and VVPA level [20], classification of PA in the higher intensities when using the AG method is more or less sorted by random chance. The misclassification is assumed to be present within this study as well, making interpretation of the PA results from the AG method questionable. For example, Fig. 1d in the present study showed that the largest relative difference between adolescent patients and controls was found at higher PA intensities using the AG method compared to using the FEM shown in Fig. 1c. The AG method contributes to an inconsistent overestimation of PA by the AG method at higher PA intensities [20], which is assumed to be more in the controls than in the patients.

In addition, the larger inter-individual variation in PA observed with the FEM is explained by that its wider filter range captures more of the variation in PA [19]. Similarly, the switch in difference between adolescent patients and controls at the highest PA intensity spectra with the FEM (Fig. 1c) is explained by the marked decrease in the proportion of individuals with recorded data (Fig. 2), which would lead to skewed distribution. The influence would be more pronounced with a frequency filter allowing more of the acceleration signal to be recorded. This reduction in the proportion of individuals having data at higher PA intensities, with larger reduction among the patients, provides information about the behavior pattern variation we seek to detect. At the same time, it makes it a challenge to perform statistical analyses across the whole PA intensity spectrum. As an example of the impact, it has previously been shown that the association with health weakened at the PA intensity level where 50% or less of the individuals provided data [22].

Physical activity is crucial for children’s normal development and health [3]. Most patients and controls in the two age-groups tended to meet the recommended amount of PA in the present study. However, conclusions from PA data collected with accelerometers in relation to the PA recommendations need to be drawn with caution, as they are based on different, not directly comparable methods. Further, the choice of cut-point for MVPA (lower cut-point, more MVPA) and the epoch-length of accelerometer data (shorter epoch-length, more MVPA) affect the proportion reaching the recommended amount of PA [4, 17]. For example, Voss et al. used 15-s epochs and a higher cut-point for MVPA than in our study when investigating children and adolescents 8–18 years with CHD [24]. We used 3-s epochs and a lower cut-point for MVPA was achieved by using the VO_2_net calibration in order to reach a reference measure of metabolic effort equivalent by age. Consequently, only 8% of the participants in their study reached the recommended amount of PA compared to 54–93% in our study. In addition, adherence to the PA recommendation also depends on the interpretation of the recommendation as ≥ 60 min daily on average (less strict) or on most days (stricter). Voss et al. applied the stricter interpretation (6 out of 7 days), which we also included in our study. Interestingly, the proportion reaching recommended PA (using the stricter criterium) was lower in the adolescents compared to the children, which is in line with the observed global trend of decreasing PA level across childhood into adolescence [26].

An amount of > 14.2 min day^−1^ less MVPA in the adolescent patient group was observed with the FEM, with approximately 5.4 min day^−1^ within the VPA + VVPA spectra where the greatest cardiometabolic health effects appear to be found [4, 22]. Although not statistically significant, these differences might still be substantial. A study by Ekelund et al. in healthy children and adolescents showed that 10 min difference in MVPA per day was associated with a 0.5 cm difference in waist circumference and a 1 pmol L^−1^ difference in fasting insulin [27]. Still, there are contradictory results regarding the cardiometabolic risk in patients with CHD. While Dean et al. reported that the metabolic syndrome was more common in adults with CHD [28], Zaquot et al. found that children with CHD did not have an increased metabolic risk [29].

Due to the relatively small sample and the novelty of the methods and findings, it is too early to draw definitive conclusions concerning the clinical implications of the present study. Children and adolescents treated for VAS may have specific limitations of performing PA [6, 9, 10]. Therefore, it is recommended that individually adapted PA determined from the clinical assessment may be prescribed (e.g., Physical Activity on Prescription), targeting also other barriers that may occur (e.g., low self-efficacy, overprotection, and restrictions from parents) [11–13]. The prescription may promote the natural and spontaneous PA pattern in children and maintenance of a physically active lifestyle including sports in adolescents. Assessment of PA may be an important part of the clinical assessment and follow-up, to provide adequate guidance, support, and feedback. New advances in the accelerometer methodology may improve PA assessment and its utility in clinical settings further.

### Limitations and Future Research

Certain limitations of the present study should be acknowledged. A larger sample would be desirable to establish the different PA patterns in children and adolescents treated for VAS. Even though all eligible patients treated for VAS were identified in the Swedish registers, there was a considerable loss of participants during recruitment. It is possible that those with less restraints and more physically active chose to participate. The loss of participants in the control group was also large. Another limitation is that gender analysis could not be performed due to the low participant number. There were also some differences in the proportion of females between the groups. These limitations may have affected the results in various ways and the identification of statistically significant differences between the groups. To be able to determine how the PA pattern in children is related to the PA later in the adolescence, a longitudinal study design would be required. Finally, with the PA being highly variable in all individuals, and probably even more inconsistent in children, the inclusion of more days than the set minimum of 4 days should be considered.

## Conclusions

Children treated for VAS had a pattern of less time in PA that may reflect short bursts of intermittent and high-intensity activity and more time in SED, while adolescents treated for VAS tended to have less time overall in PA of higher intensities and more SED, compared to their healthy peers. These patients also reported less sports participation, which supports the accelerometer results. PA assessment with the FEM displayed as a detailed spectrum of PA intensities provides more complete information regarding the PA behavior and would be implemented into future research in children and adolescents with CHD.
